# Spatial Regression and Prediction of Water Quality in a Watershed with Complex Pollution Sources

**DOI:** 10.1038/s41598-017-08254-w

**Published:** 2017-08-16

**Authors:** Xiaoying Yang, Qun Liu, Xingzhang Luo, Zheng Zheng

**Affiliations:** 1 Department of Environmental Science and Engineering, Fudan University, Shanghai, 200433 China; 2 Zhumadian City Bureau of Environmental Protection, Zhumadian, 463000 China

## Abstract

Fast economic development, burgeoning population growth, and rapid urbanization have led to complex pollution sources contributing to water quality deterioration simultaneously in many developing countries including China. This paper explored the use of spatial regression to evaluate the impacts of watershed characteristics on ambient total nitrogen (TN) concentration in a heavily polluted watershed and make predictions across the region. Regression results have confirmed the substantial impact on TN concentration by a variety of point and non-point pollution sources. In addition, spatial regression has yielded better performance than ordinary regression in predicting TN concentrations. Due to its best performance in cross-validation, the river distance based spatial regression model was used to predict TN concentrations across the watershed. The prediction results have revealed a distinct pattern in the spatial distribution of TN concentrations and identified three critical sub-regions in priority for reducing TN loads. Our study results have indicated that spatial regression could potentially serve as an effective tool to facilitate water pollution control in watersheds under diverse physical and socio-economical conditions.

## Introduction

Widespread water pollution in China has posed severe challenges towards the country’s endeavors to achieve sustainable socio-economic development and improve its people’s livelihoods^1–3^. Table 1 lists the five classes of water bodies that are specified in Chinese Surface Water Quality Standard (GB3838-2002). According to the latest 2015 Annual Report of China’s Environment Quality released by Chinese Ministry of Environmental Protection, water quality has fallen below Class III at 27.9% of the country’s 700 national routine monitoring sections along its major rivers and tributaries. Since only water bodies of class III or above can potentially serve as drinking water sources (Table 1), nearly one third of the country’s monitored stream segments are now ineligible for drinking use.

**Table 1:** Five Classes of Water Bodies Specified in Chinese Surface Water Quality Standard (GB3838-2002).

Class	Water Body Functions
I	Headwater and national nature reserves
II	First class of protected areas for centralized drinking water sources, protected areas for rare fishes, and spawning fields of fishes and shrimps.
III	Second class of protected areas for centralized drinking water sources, fishery, and swimming
IV	Industrial and recreation water use without direct human body contact
V	Agriculture and landscape

High cost of water sample collection and analysis has limited the number of routine water quality monitoring sections in China, probably also in many other countries around the world. In China, national routine monitoring sites are mostly concentrated along the main river channels and their major tributaries. For example, the Huai River basin, which drains a total area of 270,000 km^2^, has only 10 national routine monitoring sites along its main stem and an additional 42 sites along its major tributaries.

Table 2 lists the percentages of national routine monitoring river sections falling into different classes of water quality along the main stems as well as their major tributaries in China’s seven major river basins in 2015. With a larger proportion of monitoring sections falling below Class III, tributaries are generally more polluted than main stems in all seven river basins except the Hai basin. The discrepancy in the Hai basin could be partly due to the extremely limited number of monitored river sections along its main stem.

**Table 2:** Percentage of National Routine Monitoring River Sections Falling into Different Classes of Water Quality in China’s Seven Major River Basins in 2015.

Category	River Basin	Number of Monitoring Sections	Class I (%)	Class II (%)	Class III (%)	Class IV (%)	Class V (%)	Worse than Class V (%)
Main Stem	Yangtze	42	7.1	38.1	52.4	0.0	2.4	0.0
	Yellow	26	3.8	46.2	38.5	11.5	0.0	0.0
	Pearl	18	5.6	77.8	11.1	5.6	0.0	0.0
	Songhua	16	0.0	18.8	62.5	12.5	0.0	6.2
	Huai	10	0.0	30.0	50.0	20.0	0.0	0.0
	Hai	2	0.0	0.0	0.0	0.0	50.0	50.0
	Liao	14	0.0	7.1	7.1	64.3	14.3	7.1
Main Tributary	Yangtze	118	2.5	61.0	22.9	8.5	0.8	4.2
	Yellow	36	0.0	19.4	22.2	27.8	8.3	22.2
	Pearl	26	3.8	73.1	15.4	0.0	0.0	7.7
	Songhua	34	0.0	8.8	64.7	11.8	5.9	8.8
	Huai	42	0.0	7.1	28.6	26.2	21.4	16.7
	Hai	50	6.0	14.0	22.0	8.0	6.0	44.0
	Liao	6	0.0	0.0	0.0	66.7	0.0	33.3

In addition, due to their close interaction with landscape, proximity to pollution sources, and relatively limited pollutant assimilation capacity, low-order streams or minor tributaries tend to be more vulnerable than main channels to various anthropogenic disturbances such as deforestation, agriculture, and urbanization. Studies have shown that degradation of low-order streams have contributed to the water quality deterioration, such as eutrophication and hypoxia, of distant downstream ecosystems worldwide^4–6^. Up to date, routine monitoring of the water quality of the low-order streams has been usually scarce, thwarting an accurate grasp of their water quality status and timely detection of water quality issues.

Limited water quality observations, especially along the streams of lower order, may compromise our understandings of the spatial patterns of regional water quality conditions and consequently undermine the efforts to develop effective programs for watershed pollution control and water quality improvement. One feasible solution to the dilemma is to estimate regression relationships between water quality parameters and watershed characteristics based on existing water quality observations, which could be further used to identify major anthropogenic activities contributing to water pollution, assess the water quality of unmonitored stream segments, and locate crucial pollution contribution zones^7–9^.

Previous studies have examined the impacts of a variety of watershed characteristics on stream quality, such as land use and land cover, geological conditions, soil properties, topography, climate, extent of impervious surface, population density, road density, urbanization pattern, and various landscape metrics^10–18^. Many studies have adopted the ordinary regression method to determine the significant influencing factors of ambient water quality conditions and estimate the magnitude of their impacts^19–28^. Nevertheless, the ordinary regression method requires observations to be independent both spatially and temporally, which is hard to fulfill due to the potential spatial correlation between water quality samples^29^.

Spatial regression, which could incorporate the spatial correlation structure among observations into the estimation of regression coefficients, is a potential alternative for evaluating the impacts of watershed characteristics on ambient water quality conditions^30^. Up to date, however, there have been a limited number of studies exploring the use of spatial regression to investigate the impacts of watershed characteristics on water quality, most of which were conducted in agricultural watersheds where agricultural runoff has predominant impact on water quality^9, 29, 31, 32^.

Fast economic development, burgeoning population growth, and rapid urbanization have led to complex pollution sources contributing to water quality deterioration in many developing countries including China. In many watersheds of China, water bodies are simultaneously receiving a large amount of pollutant loads from multiple point and non-point pollution sources such as agriculture, rural domestic households, scattered and concentrated animal feeding operations, industries, and municipal sewage treatment plants^33–36^. Whether regression methods, especially the spatial regression method, are applicable to watersheds with such complex pollution source composition are yet to be investigated. To fill in the gap, the objectives of this study include (1) estimating the ordinary and spatial regression relationships between stream total nitrogen (TN) concentrations and watershed characteristics in a heavily-polluted watershed with complex pollution sources; (2) comparing the performance of the ordinary and spatial regression methods in predicting TN concentrations through cross-validation; and (3) making predictions of TN concentrations for unmonitored stream segments and characterizing the spatial patterns of regional TN concentration distribution.

## Results and Discussion

### Land Use and Land Cover (LULC) and Soil Distribution

**Figure 1:**
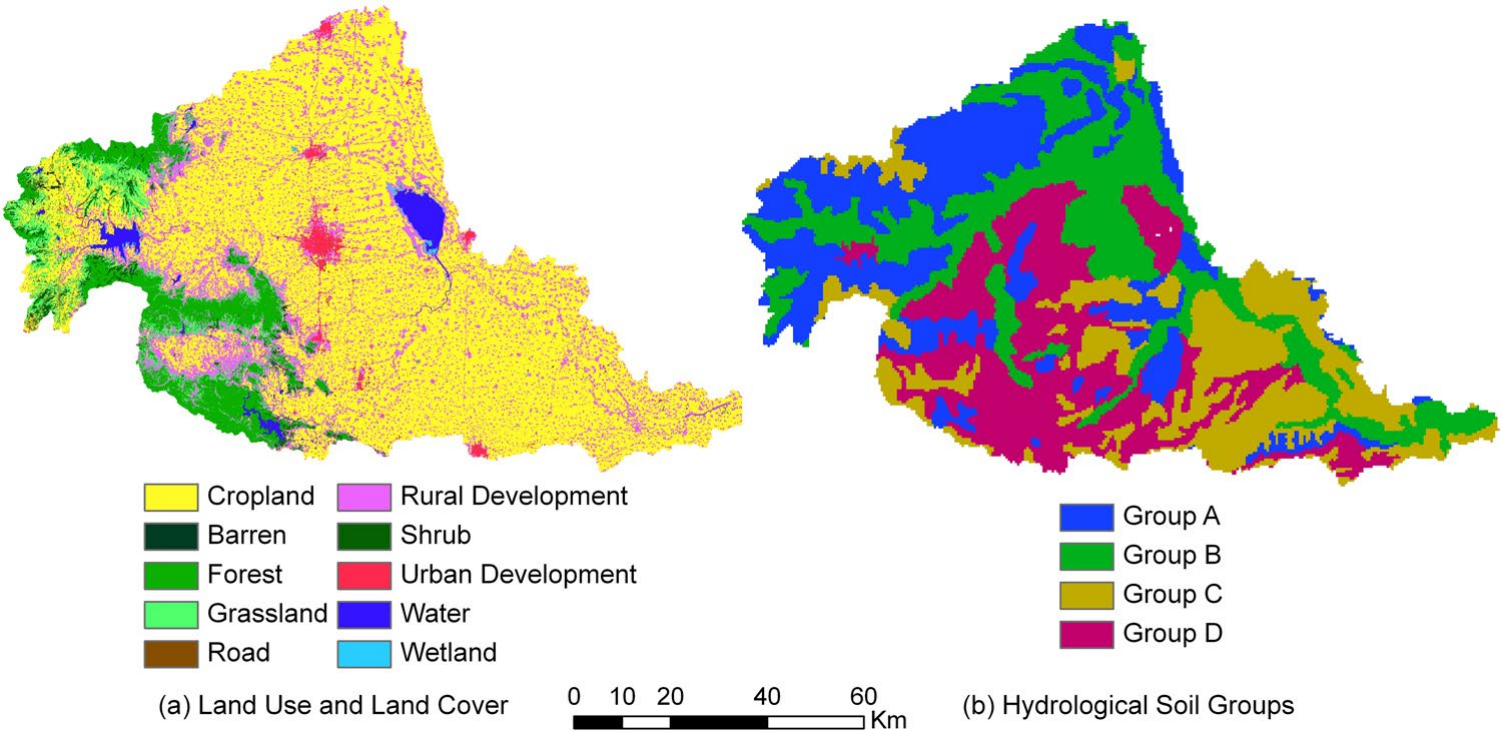
Spatial distribution of LULC and hydrological soil types in the Ru River Watershed: (**a**) Land use and land cover; (**b**) Hydrological soil groups (created by ArcGIS 9.3, http://www.esri.com/software/arcgis/arcgis-for-desktop).

**Figure 2:**
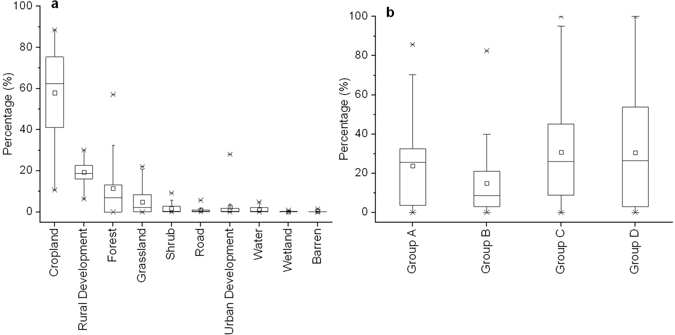
Boxplots of LULC and hydrological soil group composition in the Ru River Watershed: (**a**) Land use and land cover; (**b**) Hydrological soil groups.

The Ru River Watershed is mainly an agricultural watershed, where cropland, rural development land, forest, and grassland account for 63.1%, 18.2%, 7.8%, and 4.3%, respectively. All of the remaining six LULC types account for less than 2%. Cropland and rural development land is distributed throughout the watershed, while forest and grassland are mostly concentrated in the upstream region (Fig. 1). Among the 41 sub-watersheds, LULC composition exhibits considerable variations. For example, the percentage of cropland ranges from 10.7% to 88.4% with a standard deviation (SD) of 22.4%; the percentage of rural development land ranges from 6.3% to 30.0% with a SD of 5.6%; and the percentage of forestland ranges from 0% to 57.1% with a SD of 14.9% (Fig. 2).

All four hydrological soil groups are present in the study region. There is no single hydrological soil group in dominance since each group accounts for less than 30% of the coverage. Spatially speaking, group A and group D soils are relatively concentrated in the upstream region, while Group C soil in the downstream (Fig. 2). Like LULC, hydrological soil group composition varies greatly among the sub-watersheds. For example, the percentage of hydrological soil group A ranges from 0% to 85% with a SD of 23%, while group C ranges from 0% to 100% with a SD of 27.6% (Fig. 2).

### Regression Results

In the ordinary stepwise regression, four significant influencing factors of TN concentration were identified: percentage of cropland, distance-weighted TN load from point sources, and rural population density all with a positive coefficient; and percentage of Group C soil with a negative coefficient (Table 3). The inclusion of the three explanatory variables with positive coefficients in the stepwise regression model confirmed the considerable impact on TN concentration from a variety of point and non-point sources including agricultural production, rural domestic wastewater discharge, industrial production, and municipal sewage treatment plants. In addition, since Group C soil has relatively high runoff potential, its inclusion with a negative coefficient indicated an important role of excessive streamflow in diluting TN loads and reducing TN concentrations.

**Table 3:** Regression Coefficient Estimates of the Ordinary and Spatial Regression Models.

Parameters	Ordinary Stepwise Regression	Spatial Regression	
		Straight-line Distance	River Distance
Intercept	−7.103	−0.837	−3.118
Percentage of cropland	0.013	0.019	0.019
Percentage of hydrological soil group C	−0.018	−0.020	−0.023
Ln of distance-weighted TN load from point sources	0.237	0.287	0.262
Ln of rural population density	0.956	0.132	0.454
*θ*_1_		0.0	0.08
*θ*_2_ (km)		205	250

After stepwise regression, two spatial regression models, which were respectively based on the straight-line and river distance, were estimated. River distance was calculated as the shortest distance between monitoring sites along the stream networks. In spatial regression, only those four explanatory variables selected during ordinary stepwise regression were included. As seen from Table 3, all four explanatory variables retained the same sign in spatial regression as those in ordinary stepwise regression.

Nugget (*θ*_1_) and correlation distance (*θ*_2_), which are used to characterize the spatial correlation structure among the observations, are two new parameters specific to the spatial regression models. In both spatial regression models, the small estimates of *θ*_1_ indicated that there was little variation in stream TN concentrations over short distance in the study region. Meanwhile, the large estimates of *θ*_2_ suggested that TN concentration was spatially correlated over a long distance in the region. This may be due to the fact that nonpoint pollution sources such as agricultural runoff and rural domestic sewage discharge could affect long stretches of rivers simultaneously^37^.

### Cross-Validation

Cross-validation was used to compare the performance in predicting TN concentrations between the ordinary stepwise regression model and two spatial regression models. During cross-validation, TN concentration at each monitoring site was sequentially estimated based on TN concentrations at 8 adjacent monitoring sites. Correlation between observed and estimated TN concentrations at the 41 monitoring sites served as an indicator of model performance.

**Figure 3:**
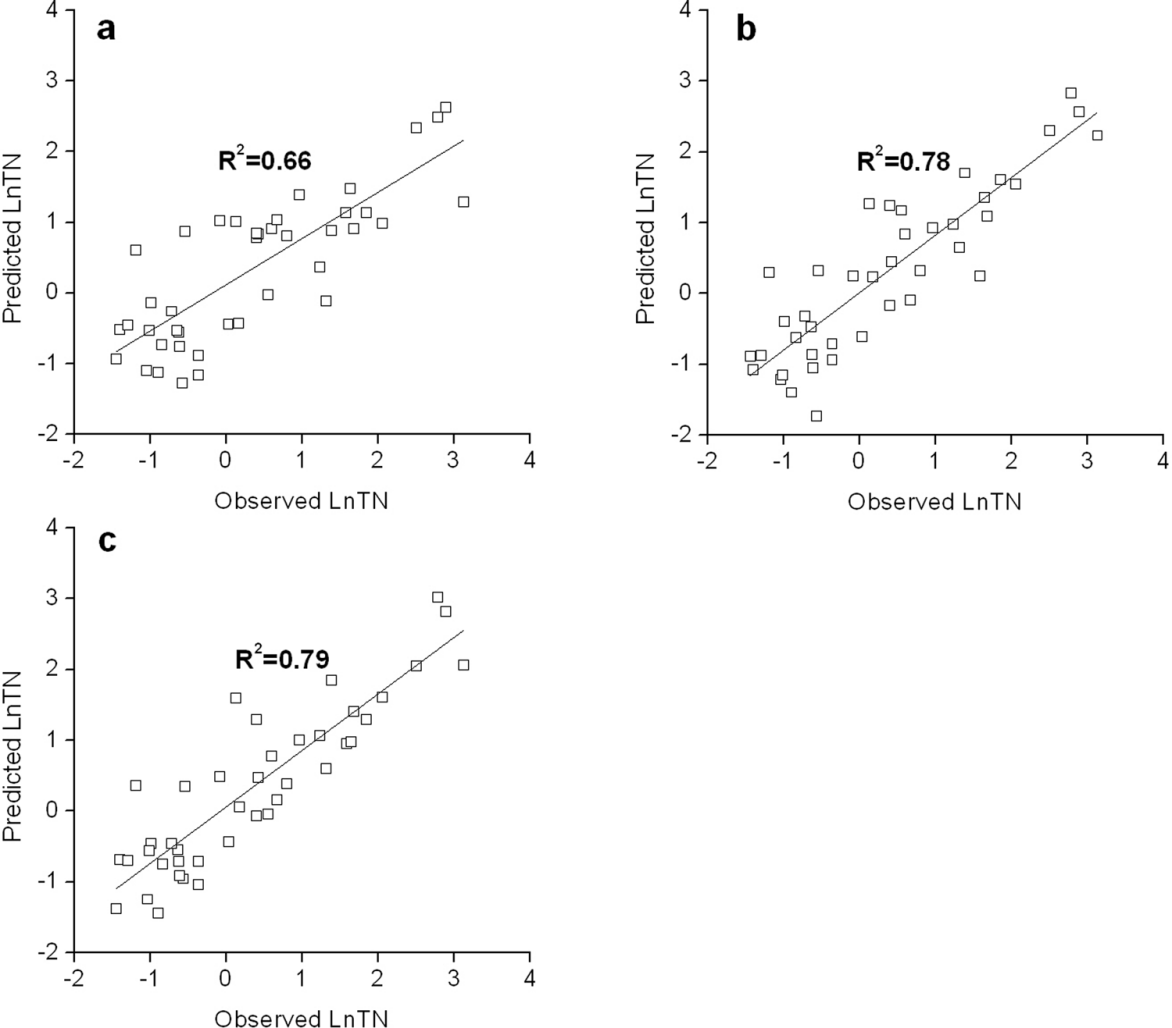
Predicted versus observed values of LnTN during cross-validation: (**a**) Ordinary stepwise regression; (**b**) Straight-line distance based spatial regression; (**c**) River distance based spatial regression.

Figure 3 compared the scatter plots of observed versus predicted values of LnTN by the ordinary stepwise regression model and two spatial regression models. The trend line and R^2^ value were also shown in each scatter plot. As seen from the figure, R^2^ increased from 0.66 in the ordinary stepwise regression model to 0.78 and 0.79 in the straight-line distance and river distance based spatial regression models, respectively. Meanwhile, the root mean square error (RMSE) decreased from 0.74 in the ordinary stepwise regression model to 0.60 and 0.58 in the two spatial regression models, respectively. This suggested that both spatial regression models performed better in making predictions of TN concentrations than the ordinary regression model. One possible reason for the better performance of spatial regression models is their capability to incorporate the spatial correlation structure in the prediction of TN concentrations. Consequently, compared to ordinary regression, spatial regression could utilize additional information such as TN concentration observations at the nearby monitoring sites for making predictions (Equation 8 and Equation 9). In the meantime, there was not much difference in the performance of the two distance measures in spatial regression.

### Spatial Prediction

**Figure 4:**
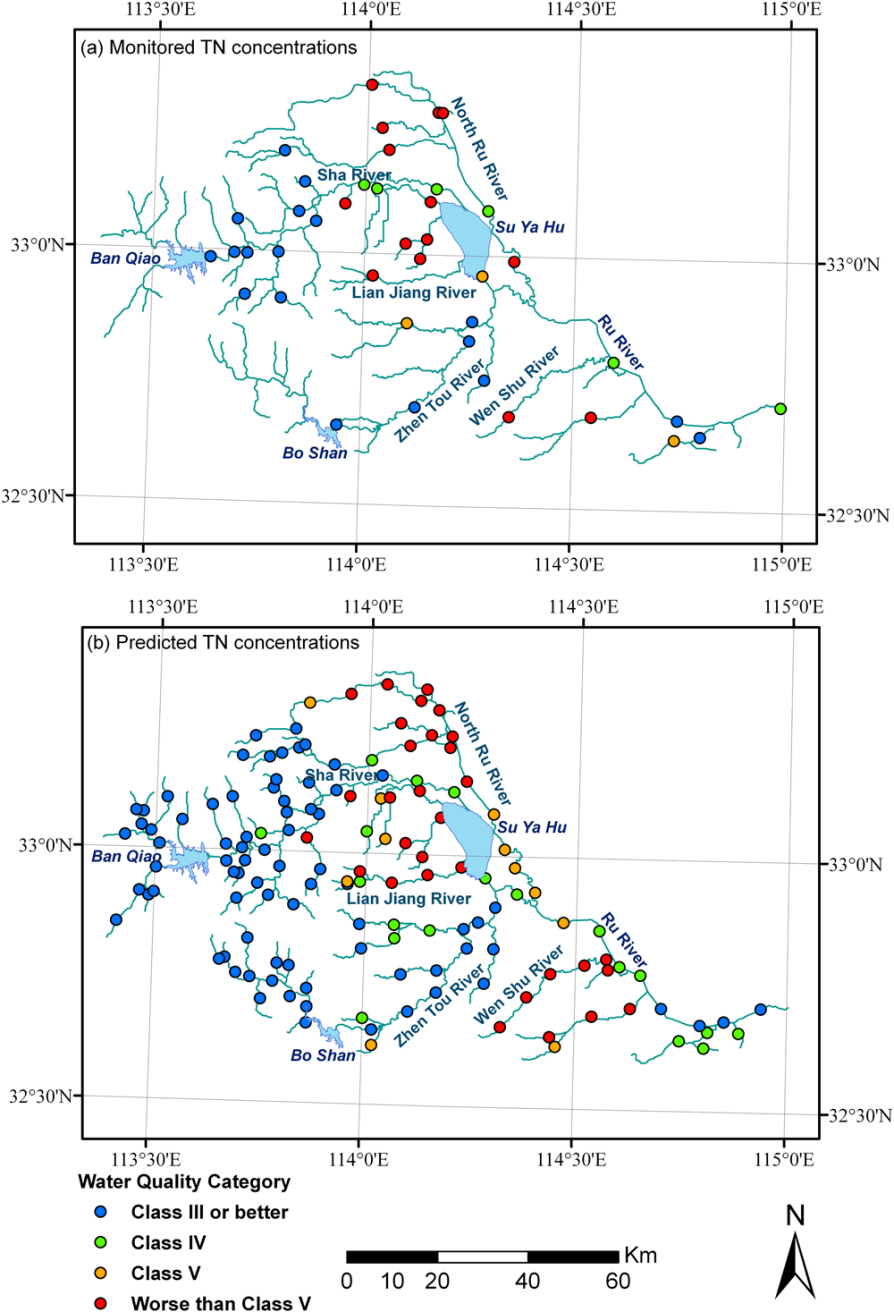
Observed versus predicted water quality categories based on TN concentrations: (**a**) Water quality categories based on monitored TN concentrations; (**b**) Water quality categories based on predicted TN concentrations with the river distance based spatial regression model (created by ArcGIS 9.3, http://www.esri.com/software/arcgis/arcgis-for-desktop).

The Ru River Watershed is one of the most severely polluted sub-basins in the Huai River Basin. However, only four sites are being routinely monitored by the local Environmental Protection Agency in the watershed, three of which are located in the three reservoirs (Ban Qiao, Bo Shan, and Su Ya Hu) and one other site located below the confluence of the Sha River and Zhen Tou River (Fig. 4). Limited water quality monitoring makes it hard to comprehend the spatial distribution of water pollution in the region as well as pinpoint the critical areas that are in priority for reducing pollutant loads.

Local authorities are especially concerned about the number and the spatial distribution of stream segments whose water quality falls below Class V since they are virtually not suitable for any use (Table 1). According to GB3838-2002, water quality of a stream segment is classified to be worse than Class V if its TN concentration exceeds 2 mg/L. Table 4 compared the performance of the three models in classifying the 41 monitoring sites into two categories based on their predicted TN concentrations during cross-validation: *Class V or better* and *worse than Class V*. The river distance based spatial regression model made 36 correct classifications, compared to 34 by the straight-line distance based spatial regression model and 31 by the ordinary regression model. Due to its better performance in classification, the river distance based spatial regression model was chosen to further make predictions of TN concentrations for all stream segments throughout the Ru River Watershed.

**Table 4:** Comparison of Performance in Water Quality Category Classification Based on TN Concentrations.

Category		Ordinary Regression Model	Spatial Regression Model	
			Straight-line Distance	River Distance
Correct Classification		31	34	36
Wrong Classification	Classify *V or better* as *worse than V*	8	4	3
	Classify *worse than V* as *V or better*	2	3	2

To make predictions of TN concentrations throughout the Ru River Watershed, a total of 146 sites, which are 10 km apart, were generated along the main stem and tributaries of the Ru River. The river distance based spatial regression model was then utilized to predict TN concentrations at the 146 generated sites. In making predictions, the upstream contribution area to each generated site was first delineated and its values of four significant influencing watershed characteristics were calculated (Table 3). TN concentration at each generated site was then estimated using Equations (8) and (9) based on the residuals at 8 adjacent monitoring sites.

Among the 146 generated sites, TN concentrations at 32 sites were predicted to fall below Class V, 12 sites in Class V, 19 sites in Class IV, and 83 sites in Class III or above. Although the watershed was known to be heavily polluted, our prediction results have shown that the spatial distribution of TN concentrations in the area was far from being uniform but exhibited distinctive spatial patterns. With the majority of their sites falling in Class III or above, water quality in the upstream of the Sha River and Zhen Tou River was relatively good. On the other hand, both the upstream North Ru River and the downstream Wen Shu River were seriously polluted, whose sites all fell in Class V or worse. In addition, the tributaries to the Su Ya Hu reservoir were also severely polluted, with the majority of their sites falling in Class V or worse. The revealed substantial spatial disparity in TN concentrations suggested that a differentiated approach, which put the priority of TN load reduction in the three severely polluted sub-regions (i.e. the upstream of the Sha River, the upstream of the Zhen Tou River, and the tributaries to the Su Ya Hu reservoir), may be more cost-effective in improving the overall water quality of the watershed (Fig. 4).

## Conclusions

Widespread water pollution has posed severe challenges towards sustainable development in many developing countries including China. In stark contrast to the widespread water pollution is their limited coverage of water quality monitoring networks. The lack of effective monitoring is especially serious in the low-order stream segments or minor tributaries, which unfortunately tend to be more vulnerable to human disturbances. Sound knowledge of water quality conditions is the prerequisite to developing effective watershed pollution control programs. Estimating regression relationships between water quality parameters and significant influencing watershed characteristics has proved to be an effective approach to amend the deficiency in water quality observations and facilitate sound decision-making in watershed pollution control.

In view of the potential spatial correlation between water quality observations, this study explored the use of spatial regression in the Ru River Watershed, one heavily polluted headwater region of the Upper Huai River Basin in China. To develop the regression model, water quality observations were made at 41 sites along both the main stem and tributaries of the Ru River. Regression results have shown that TN concentrations are much affected by human activities as well as physical properties of the watershed. Crop production, industrial activity, and domestic wastewater discharge are the main sources contributing to N pollution in the region. Composition of hydrological soil groups, which directly affects the migration of N from land to streams, is also a significant influencing factor.

Comparison between the ordinary stepwise regression model and the two spatial regression models has indicated a better performance by spatial regression in predicting TN concentrations. With the best prediction performance during cross-validation, the river distance based spatial regression model was used to predict TN concentrations across the Ru River Watershed. The prediction results have revealed a distinct pattern in the spatial distribution of TN concentrations and identified the following three critical sub-regions for reducing TN loads: the upstream of the Sha River, the upstream of the Zhen Tou River, and the tributaries to the Su Ya Hu reservoir.

To our knowledge, this study is the first attempt to use spatial regression to investigate the impacts of complex pollution sources on ambient water quality. This study, along with a limited number of published studies, has demonstrated that spatial regression modeling could potentially serve as an effective tool to facilitate water pollution control in watersheds under diverse physical and socio-economical conditions. It is suggested that similar studies should be conducted in watersheds under a variety of natural and man-made settings so as to fully evaluate the robustness of the performance of spatial regression models. More efforts are also needed to complement the results of spatial regression with those from process-based watershed models to gain an in-depth understanding of the movement of water pollutants in the region and formulate effective water pollution control programs.

## Methods

### Study Region

Located in eastern China between the Yangtze River and the Yellow River, the Huai River (111°55′–121°25′E and 30°55′–36°36′N) drains a total area of 270,000 km^2^ with a population of 165 million. With a population density of nearly 5 times the national average, the river basin is one of the most densely populated regions in China. Meanwhile, it has been one of the most polluted river basins in China due to its fast economic development and lack of effective control of sewage discharge from industries, households, and other sources. According to the latest 2015 Annual Report of China’s Environment Quality, water quality at nearly half of its 94 national routine monitoring sections was assessed to be Class IV or worse. The tributaries of the Huai River were even more polluted than its main reach, with around 65% of their routine monitoring sections falling into Class IV or worse.

The Ru River is one tributary to the upstream Huai River in Henan Province, which has been facing the severe challenge of serious water pollution. Originating from the Ban Qiao Reservoir, the river flows 223 km mostly southeast through the Sui Ping, Ru Nan, Ping Yu, and Xin Cai Counties of the Zhu Ma Dian City before pouring into the Hong River, which ultimately joins the upstream Huai River.

With hills in the west and plains in the east, surface elevation in the Ru River Watershed ranges from 34 m to 952 m. Located in a transition zone between the northern subtropical and warm temperate climate, local climate features four distinctive seasons with an annual mean temperature of around 15 °C. Its annual precipitation typically fluctuates between 860 mm and 980 mm, most of which occurs in the summer months from June to August.

### Water Quality Monitoring and Sample Analysis

**Figure 5:**
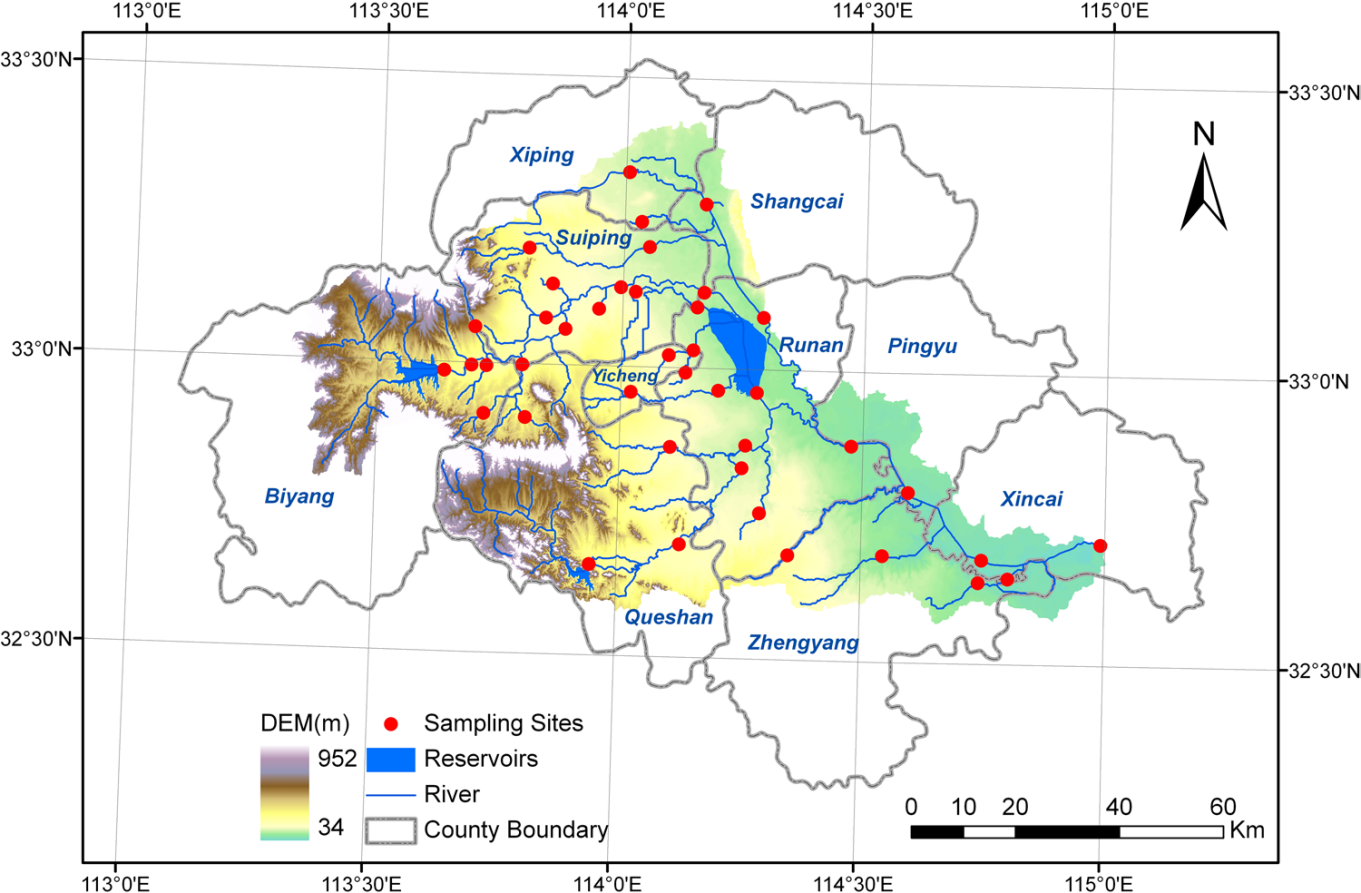
Study region (created by ArcGIS 9.3, http://www.esri.com/software/arcgis/arcgis-for-desktop).

A “snapshot” monitoring campaign was conducted along the Ru River and its tributaries, covering a total area of around 7335 km^2^. Water samples at 41 sites along both the main stem and tributaries of the Ru River were collected within three days (Fig. 5). At each site, duplicate grab samples were collected from the middle of the stream at 0.5 m below the water surface using a 1000-ml organic glass hydrophore. After being transferred to polyethylene bottles and acidified with sulfuric acid, the water samples were immediately sent to the local environmental monitoring station for chemical analysis. Storage, preservation and chemical analysis all followed the guidelines given by the former Chinese Bureau of Environmental Protection^38^.

### Influencing Factors of Water Quality

Four categories of watershed characteristics were included in the regression models to evaluate their impacts on stream TN concentrations (Table 5). The 25 m Digital Elevation Model (DEM) data was obtained from the National Geomatics Center of China, which was used to delineate the upstream contribution area of each water sampling site (sub-watershed) and calculate its topographical characteristics such as mean slope and total area. Distribution of ten land use and land cover (LULC) types of the study region was derived through the supervised classification of the Landsat-TM images of 2013 based on field survey results. LULC composition of each sub-watershed was then calculated.

**Table 5:** Potential Influencing Factors of TN Concentrations.

Category	Explanatory Variables
Topography	Mean slope
	Area
Land use and land cover	Percentage of cropland
	Percentage of forestland
	Percentage of grassland
	Percentage of urban development land
	Percentage of rural development land
	Percentage of wetland
	Percentage of shrub
	Percentage of barren land
	Percentage of road
	Percentage of water
Soil property	Percentage of hydrological soil group A
	Percentage of hydrological soil group B
	Percentage of hydrological soil group C
	Percentage of hydrological soil group D
Pollution source	Distance-weighted TN load from point sources
	Rural population density
	Animal manure load density

Spatial distribution of soil types and their properties were obtained from Nanjing Institute of Soil Science^39–42^. Soils were further classified into four hydrologic soil groups based on their runoff potential: A, B, C, and D^43^. Group A soils have the lowest runoff potential, while Group D soils have the highest potential. Proportions of four hydrological soil groups in each sub-watershed were calculated.

Multiple point and non-point pollution sources contribute TN loads to the Ru River including industries, municipal sewage treatment plants, animal feeding operations, crop production, and rural households. Annual TN loads from industries were extracted from the database of 2010 Census of Pollution Sources in the Zhu Ma Dian City. Annual TN discharge from six municipal sewage treatment plants were obtained from the Bureau of Environmental Protection of Zhu Ma Dian City. In each sub-watershed with multiple industries and/or municipal sewage treatment plants, a composite point pollution source was created with its TN load equal to the sum of the loads from all industries and municipal sewage treatment plants in the sub-watershed. A variable called “distance-weighted TN load from point sources” (*TN_PointSource_DW*) was created to evaluate the impact of point sources on TN concentration at each water sampling site:

1 $$TN\_Po\,\mathrm{int}\,Source\_DW=\sum _{i=1}^{n}\frac{T{N}_{i}}{{D}_{i}}$$

Where n is the number of upstream sub-watersheds with a single or composite point pollution source; D_i_ is the river distance between the point pollution source in sub-watershed i and the water sampling site; TN_i_ is the TN load of the point pollution source in sub-watershed i.

Like many other regions in China, rural domestic sewage has not been collected for central treatment in the study region. Rural population density of each sub-watershed was calculated to evaluate the impact of rural sewage on ambient TN concentrations. Rural population of the nine counties and one district located fully or partially in the study region was first obtained from the Statistical Yearbook of Zhu Ma Dian City. Rural population density of individual sub-watershed was then estimated as the area-weighted average of county/district rural population density.

County level data on total amount of animal manure from animal feeding operations were obtained from the Bureau of Animal Husbandry of Zhu Ma Dian City, while county level data on cropland acreage from the Statistical Yearbook of Zhu Ma Dian City. Based on the two, animal manure load density in each country could be estimated. Animal manure load density of individual sub-watershed was then calcuated as the area-weighted average of county level animal manure load density.

### Regression Model Estimation

#### Stepwise regression model

Ranging from 0.2 to 22.6 mg/l with a mean of 3.3 mg/L, TN concentration falls below Class III at 56.1% of the 41 sampling sites and below Class V at 34.1% of the sites. Due to its skewed distribution, a logarithmic transformation was applied to TN concentration before its being used in regression estimation as in many previous studies^44–47^. Minitab 16.0 was then used to conduct an ordinary stepwise regression to identify the significant explanatory variables that could explain the variation in TN concentrations. Like any ordinary least square (OLS) regression model, the selected variable ensemble should not violate the preconditions such as avoiding multicollinearity and heteroscedasticity^48^.

#### Spatial regression model

Unlike ordinary stepwise regression, spatial regression is not designed to identify significant explanatory variables, but to reduce the potential bias in the ordinary regression coefficient estimates by incorporating the spatial correlation among regression residuals. In this study, spatial regression models were estimated only with the significant (α = 0.05) explanatory variables chosen during the ordinary stepwise regression. The spatial regression model takes the following form^49^:

2 $$Y=X\beta +\varepsilon $$

where *Y* is the vector of the dependent variable; *X* is the $n\times p$ matrix on the intercept term plus $(p-1)$ explanatory variables; *β* is the vector of *p* regression coefficients; *ε* is the vector of *n* residual terms that are spatially correlated.

Unlike the ordinary regression model, spatial regression models assume residuals (*ε*) are spatially correlated following a normal distribution with zero mean and a variance-covariance matrix of *σ* ^2^ *Ω*, where *σ* ^2^ is the variance and *Ω* (*d*; *θ*_1_; *θ*_2_) is the correlation matrix. The correlation matrix was estimated using the following exponential auto-correlation function:

3 $$C(d;{\theta }_{1};{\theta }_{2})=\{\begin{array}{ll}1, & d=0\\ (1-{\theta }_{1})\exp (-d/{\theta }_{2}), & d > 0\end{array}$$

Where *d* is distance between monitoring sites; *θ*_1_ is the proportion of nugget effect; and *θ*_2_ is the range parameter. In spatial regression, distance between observations is used to indicate their similarity. Different distance measures have been proposed. The most commonly used distance measure is the straight-line distance. However, it may not function well with stream monitoring sites since it fails to incorporate the connectivity and topology of stream networks. Hydrological distance, which measures the distance between stream monitoring sites along the stream networks, has been proposed as an alternative for studying their spatial correlation^50–52^.

The maximum likelihood method was used to estimate three categories of parameters in the spatial regression model: the regression coefficient vector *β*, the spatial correlation structure parameters *θ*_1_ and *θ*_2_, and the variance *σ* ^2^. The log-likelihood function of the parameters (*θ*_1_ *, θ*_2_ *, β, σ* ^2^) with respect to the dependent variable *Y* is:

4 $$l({\theta }_{1},{\theta }_{2},\beta ,{\sigma }^{2};Y)=-\frac{n}{2}\,\mathrm{log}(2\pi )-\frac{1}{2}\,\mathrm{log}|{\sigma }^{2}{\rm{\Omega }}|-\frac{1}{2{\sigma }^{2}}(Y-X\beta )^{\prime} {{\rm{\Omega }}}^{-1}(Y-X\beta )$$

Maximizing the log-likelihood function in Equation (4) with respect to *β* and *σ* ^2^ yielded their maximum likelihood estimators:

5 $$\hat{\beta }={(X^{\prime} {{\rm{\Omega }}}^{-1}X)}^{-1}X^{\prime} {{\rm{\Omega }}}^{-1}Y$$

and

6 $${\hat{\sigma }}^{2}=\frac{(Y-X\hat{\beta })^{\prime} {{\rm{\Omega }}}^{-1}(Y-X\hat{\beta })}{n}$$

Substituting the maximum likelihood estimators $\hat{\beta }$ and ${\hat{\sigma }}^{2}$ in Equations (5) to (6) into Equation (4) produced the profile log-likelihood function:

7 $${l}_{profile}({\theta }_{1},{\theta }_{2};\hat{\beta },{\hat{\sigma }}^{2},Y)=-\frac{n}{2}\,\mathrm{log}(2\pi )-\frac{n}{2}\,\mathrm{log}({\hat{\sigma }}^{2})-\frac{1}{2}\,\mathrm{log}|{\rm{\Omega }}|-\frac{n}{2}$$

Maximizing the profile log-likelihood function in Equation (7) yielded the estimates of *θ*_1_ and *θ*_2_, which were then used to estimate β and σ^2^ with Equations (5) and (6). Contours of the profile likelihood value against the two unknown parameters (*θ*_1_ and *θ*_2_) were plotted to verify the optimization results.

### Spatial Prediction

The estimated spatial regression model could be utilized to make predictions of TN concentrations at unmonitored locations. TN concentration at certain unmonitored location (denoted as location 0) could be estimated as:

8 $${\hat{Y}}_{0}={X}_{0}\mathop{\beta }\limits^{\frown {}}+{\hat{\varepsilon }}_{0}$$

where: ${\hat{Y}}_{0}$ is TN concentration at location 0; *X*_0_ is the vector of 1 plus the values of $(p-1)$ significant explanatory variables at location 0; $\hat{\beta }$ is the vector of *p* estimated spatial regression coefficients; and ${\hat{\varepsilon }}_{0}$ is the regression residual at location 0, which was estimated based on the regression residuals at the nearby monitoring sites by ordinary kriging:

9 $${\hat{\varepsilon }}_{0}=\sum _{i=1}^{m}{\lambda }_{i}\,{\hat{\varepsilon }}_{i}$$

Where *m* is the number of nearby monitoring sites included in ordinary kriging; ${\hat{\varepsilon }}_{i}$ is the regression residual at monitoring site *i*; and *λi* is the ordinary kriging weight of monitoring site *i*. The exponential auto-correlation function estimated during spatial regression (Equation (3)) was used to derive the ordinary kriging weights.
